# Estimation of Knee Extension Force Using Mechanomyography Signals Based on GRA and ICS-SVR

**DOI:** 10.3390/s22124651

**Published:** 2022-06-20

**Authors:** Zebin Li, Lifu Gao, Wei Lu, Daqing Wang, Huibin Cao, Gang Zhang

**Affiliations:** 1 Institute of Intelligent Machines, Hefei Institutes of Physical Science, Chinese Academy of Sciences, Hefei 230031, China; lifugao@iim.ac.cn (L.G.); dqwang@mail.ustc.edu.cn (D.W.); hbcao@iim.ac.cn (H.C.); 2 Department of Science Island, University of Science and Technology of China, Hefei 230026, China; 3 School of Electrical and Photoelectric Engineering, West Anhui University, Lu’an 237012, China; zhanggang@wxc.edu.cn

**Keywords:** muscle force estimation, MMG, gray relational analysis, machine learning, improved cuckoo search algorithm

## Abstract

During lower-extremity rehabilitation training, muscle activity status needs to be monitored in real time to adjust the assisted force appropriately, but it is a challenging task to obtain muscle force noninvasively. Mechanomyography (MMG) signals offer unparalleled advantages over sEMG, reflecting the intention of human movement while being noninvasive. Therefore, in this paper, based on MMG, a combined scheme of gray relational analysis (GRA) and support vector regression optimized by an improved cuckoo search algorithm (ICS-SVR) is proposed to estimate the knee joint extension force. Firstly, the features reflecting muscle activity comprehensively, such as time-domain features, frequency-domain features, time–frequency-domain features, and nonlinear dynamics features, were extracted from MMG signals, and the relational degree was calculated using the GRA method to obtain the correlation features with high relatedness to the knee joint extension force sequence. Then, a combination of correlated features with high relational degree was input into the designed ICS-SVR model for muscle force estimation. The experimental results show that the evaluation indices of the knee joint extension force estimation obtained by the combined scheme of GRA and ICS-SVR were superior to other regression models and could estimate the muscle force with higher estimation accuracy. It is further demonstrated that the proposed scheme can meet the need of muscle force estimation required for rehabilitation devices, powered prostheses, etc.

## 1. Introduction

The central nervous system quantitatively controls force production of the skeletal muscles through the successive recruitment of motor units (MUs) [1]. These forces contribute to the generation of the forces required to perform various movements and to interact with the external environment. The skeletal muscles, as the power source of the motor system, work together with the bones and joints to accomplish basic limb movements such as standing, sitting, walking, and jumping, as well as complex movements, under the innervation of the nervous system. Although these behaviors do not directly affect people’s survival, they are directly related to living independently and autonomously.

In the real world, common muscular and neurological disorders, such as stroke and hemiplegia, as well as cumulative diseases such as arthritis, lead to movement problems in the skeletal muscle system and severely affect the body’s ability to move freely [2]. Rehabilitation with assistive devices has received increasing attention for helping these patients regain their ability to live independently and improve their standard of living.

Some rehabilitation auxiliary devices in structured environments are able to obtain kinematic information about the human body during limb movement through sensors or the mechanical principle of the device itself to achieve simple repetitive tasks [3]. However, these devices in unstructured environments should be augmented with force-sensing functions to improve flexibility and interaction capabilities; otherwise, they will fail.

Currently, the force control and interaction capabilities of these assistive devices lag far behind the expected results [4,5] and do not reflect the user’s movement intention, much less operate flexibly. Most patients do not receive satisfactory active training needs, which plays a key role in the rehabilitation process. Therefore, it is necessary to accurately predict and estimate the human muscle force, which helps these auxiliary devices to not only improve their naturalness and flexibility, but also respond to and assist the user in a timely and appropriate manner [6,7]. Accordingly, their users can receive effective and comfortable training.

The knee joint of the lower limbs is one of the most widely studied objects in rehabilitation, and it determines the movement of the human lower limb. Knee joint force plays a crucial role in completing lower-limb movement and interacting with the external environment. The knee joint force is a biomechanical measure used to infer structural loads on the knee joint. The timely and accurate estimation of the lower-limb force information is expected to be used to determine the output power and force information of the auxiliary equipment, as well as to determine more flexible control.

Neural information is not only complex and time-varying, but also has a nonlinear mapping relationship with human movement intention, which poses a challenge in terms of intention decoding and subsequent human–computer interaction [8]. Compared with the traditional muscle force measurement method, human surface biosignals, such as electromyography (sEMG) and mechanomyography (MMG), are easy to measure and do not damage the human body during the measurement process. Therefore, the estimation of muscle force based on human surface biosignals has received more and more attention from researchers and has been widely used in many fields, such as rehabilitation training, intelligent prosthesis control, sports medicine, and human–computer interaction [9,10].

Since the features of sEMG show a nonlinear and complex relationship with muscle force, in recent years, many researchers have started to estimate or predict human kinematic parameters with sEMG using models such as neural network models and support vector machines (SVM), obtaining fruitful results. Peng et al. [11] used two three-layer backpropagation neural networks (BPNNs) to estimate muscle torques at hip and knee joints, on the basis of sEMG, and used them in rehabilitation robots to achieve real-time coordinated active training, obtaining better results. Wang et al. [12] used a cross-model selection (CMS) technology to estimate the handgrip force on the basis of sEMG and obtained a high accuracy. Song et al. [13] used a recurrent artificial neural network (RANN) to estimate the joint torque on the basis of sEMG and also obtained a better prediction accuracy. Even though these studies demonstrated that sEMG obtains outstanding results in estimating or predicting human kinematic parameters, it is worth noting that sEMG sensors can be affected by environmental noise, skin surface cleanliness, and placement, which often lead to problems in terms of the actual test signal instability during sEMG testing. These not only bring difficulties but also further limit their practical application [14,15].

Related studies have shown that, in addition to sEMG signals, MMG signals can be generated during muscle activity, which are generated by the mechanical vibration of muscle fibers [16,17,18]. MMG signals can provide information about the number and firing rates of recruited motor units, reflecting the characteristics of muscle activity [19]. As a result, MMG signals are similar to sEMG and also contain rich information such as muscle state, movement pattern, and movement intention [20]. MMG signals can be detected by various types of sensors that do not need to be placed in precise locations and can also be placed on clothes. Additionally, since MMG signals are mechanical signals, their detection process is also not affected by the change in skin impedance [21]. Compared with sEMG, MMG signals have unparalleled advantages, have attracted extensive attention from many researchers in recent years, and have also been applied in many fields, such as prosthetic hand control [9], muscle fatigue assessment [22], human movement recognition [13], and human kinetic parameter estimation [23,24,25,26,27]. However, because of the characteristics of weak signal strength, low frequency, and strong randomness, it also poses a challenge to decode MMG signals and use them to estimate and predict muscle force. When MMG signals are used for muscle force estimation, there are two critical issues to be addressed: one is to extract the features that can effectively reflect muscle activity; the other is to construct an appropriate model for muscle force estimation.

In recent years, researchers have made valuable attempts in muscle force estimation using MMG signals. Akataki et al. [28] found the MMG/force relationship through the RMS amplitude/force and MPF/force relationships during voluntary isometric ramp contractions of the biceps brachii muscles. Beck et al. [29] found a linear increase in MMG amplitude with isokinetic torque, but no significant change relationship between MMG MPF and isokinetic torque during isokinetic muscle actions of the biceps brachii. With further research, the researchers found the presence of a nonlinear relationship between MMG and muscle force, indicating that MMG can be used to estimate muscle force. Therefore, subsequent researchers also started to extract time-domain features and frequency-domain features from MMG signals and fed them into a machine learning model to achieve a nonlinear MMG–force relationship [25,26,27]. Although some achievements have been made in muscle force estimation, there are still some drawbacks such as fewer features considered and lower accuracy of the estimated models. Recently, to improve the accuracy of muscle force estimation, our research group tried to build an optimized SVR to estimate the knee joint extension force by selecting root mean square (RMS), mean absolute value (MAV), zero crossing (ZC), mean power frequency (MPF), and sample entropy (SE). Although good effects and accuracy were achieved, there are still some shortcomings, such as inefficient models and empirical selection of features that do not fully reflect the muscle activity.

At present, the main features for characterizing MMG signals include time-domain features, frequency-domain features, and time–frequency-domain features. Due to the nonlinear chaotic nature of MMG signals [30], nonlinear dynamics features should also be considered. However, all the features are fed into a machine learning model, which will increase not only the computational complexity but also the computational burden, eventually leading to overfitting of the model and degradation of the estimation accuracy. Therefore, dimension reduction techniques, such as principal component analysis (PCA), independent component analysis (ICA), and linear discriminant analysis (LDA), are widely used to obtain effective features [31,32]. However, these methods tend to destroy the original data structure. Therefore, we need to investigate other methods of feature dimension reduction to avoid destroying the original data structure.

Support vector machine (SVM) is a machine learning method based on statistical learning theory proposed by the research group led by Vapnik in 1995 [33]. With outstanding nonlinear processing capability, high-dimensional mapping ability, and kernel computing technology, the support vector regression (SVR) model has already yielded significant and abundant application results in many fields [34,35]. However, when applying the SVR model in practice, the selection of critical parameters, such as penalty coefficient *C* and kernel function width parameter *σ*, can directly affect the prediction accuracy of the model. Usually, trial-and-error and grid search methods are used to select critical parameters, which are not only time-consuming but also ineffective.

Since Beni and Wang first proposed the concept of swarm intelligence in 1989, swarm intelligence algorithms have been continuously developed and used by many researchers to optimize and find the optimal critical parameters in machine learning models such as SVM, GRNN, and BPNN [36,37,38]. Among intelligent algorithms, the cuckoo search (CS) algorithm has attracted the attention of researchers because of its powerful optimization ability and few adjustment parameters [38]. In particular, the application of CS to SVR in many fields has achieved good effects. Yang et al. [39] used CS-SVR to evaluate and monitor the health status of radar equipment and obtained a high recognition rate. Bo et al. [40] used CS-SVM to predict the postoperative recurrence time and recurrence location of patients with liver cancer for studying the mechanism of liver cancer recurrence; the results indicated that CS-SVM could effectively predict the time and location of cancer recurrence, and the prediction results of CS-SVM outperformed those of BPNN. Although the CS algorithm has been applied in many fields with its advantages, it still has some shortcomings, such as uneven population diversity and poor adaptiveness of the search process [41]. Therefore, developing an improved CS (ICS) method to overcome these shortcomings and using ICS to optimize the critical parameters of SVR are beneficial to the solution of practical problems.

**Figure 1:**
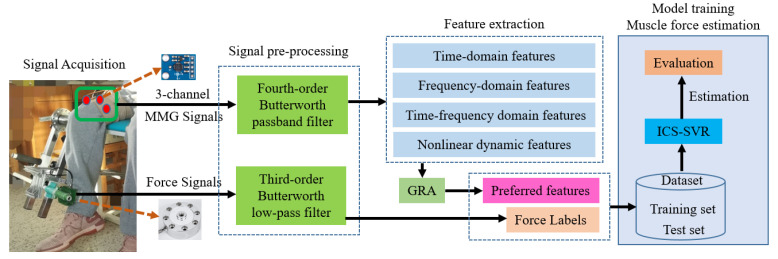
The block diagram of knee joint extension force estimation.

As the application of MMG signals in engineering technology continues to be developed, we must consider the influence of effective features reflecting muscle activity and the effects of estimation models when using MMG signals to estimate muscle force. Therefore, in this paper, the MMG feature expression and the adaptability of the model were fully considered, and a scheme based on GRA and the ICS-SVR model was proposed to estimate knee joint extension force. Figure 1 shows the specific process of knee joint extension force estimation based on the proposed scheme, via which the highly accurate estimation of knee joint extension force can be realized. The main contributions of this paper are as follows:

(1) In this paper, the features reflecting muscle activity were fully considered to avoid the limitations of a single category of features and conventional features. We extracted time-domain features, frequency-domain features, and time–frequency-domain features from MMG signals, as well as nonlinear dynamics features. In order to obtain effective features that are highly correlated with muscle force, GRA was employed for effective feature selection, aiming to achieve high accuracy muscle force estimation using these effective features reflecting muscle activity.
(2) In this paper, muscle force estimation was based on the SVR model, whose performance depends entirely on critical parameters (*C*, *σ*). The cuckoo search (CS) algorithm can optimize the SVR parameters due to the advantages of fast convergence, few parameters, and easy implementation. To obtain better optimization performance, we improved the CS algorithm using a chaotic Tent initialization population and adaptive control parameters. Compared with other optimization algorithms, the optimal global minimum and optimal convergence performance of ICS were obtained in test benchmark functions.
(3) In this paper, combining the advantages of GRA and ICS-SVR, we designed an MMG–force scheme to effectively obtain muscle activity features and accurately perform muscle force estimation.

The remainder of the paper is arranged as follows: the model building process and related algorithms are given in Section 2; signal acquisition and processing are described in Section 3; experiments and results are given in Section 4; the discussion is given in Section 5; the conclusion is described in Section 6.

## 2. ICS-SVR

### 2.1. SVR

SVM, developed by Vapnik [33], is considered one of the most powerful supervised machine learning algorithms based on the structural risk minimization principle. As an effective tool to solve certain problems in complex data processing, SVM can overcome traditional difficulties such as dimensional disaster and overlearning. Compared with neural networks, it has a reliable statistical theory foundation and higher prediction accuracy for high-dimensional small sample data, which is especially suitable for nonlinear problems and has been successfully applied in the fields of classification and prediction.

SVR is a class of support vector machines for fitting regression problems, which has excellent fitting ability, robustness, and fault tolerance for nonlinear and unstable data. On the basis of a previous study, this paper also chose the RBF function. However, the critical parameters of SVR are difficult to select using experience and trial-and-error methods [42]. Traditional parameter search methods such as grid search, particle swarm optimization algorithm (PSO), and genetic optimization algorithm (GA) have the disadvantages of falling into local optimization or slow calculation speed. Therefore, in this paper, we chose the improved swarm intelligence algorithm ICS to search for these critical parameters, so as to avoid the overfitting phenomenon caused by improper parameter selection.

### 2.2. Cuckoo Search Algorithm

CS is a novel type of heuristic algorithm used to simulate the egg-laying behavior of a cuckoo, which has been extensively applied to solve optimization problems in different fields of engineering [43]. The algorithm has many advantages such as fast convergence, high stability, few parameters, and simple operation, and it is very effective in solving global optimization because it can maintain a balance between local and global random walks using switching parameters. The algorithm is developed from the parasitic reproduction strategy of the cuckoo population itself and Lévy flight behavior. For specific practical applications, the following three ideal assumptions are made [44]:

(1) Each cuckoo lays one egg in a randomly selected host nest at a time.
(2) Following the survival of the fittest principle, a strong surviving egg among all the host nests is inherited by the next generation.
(3) For a fixed number of host nests, the probability of the intruder egg being found by the host bird is *P_a_* ∈ [0, 1].

On the basis of the above assumptions, the position of the cuckoo’s nest can be updated as follows:

(1) $$X_{i}^{t+1}=X_{i}^{t}+\alpha ⨂Lévy\left(\lambda \right),$$

where $X_{i}^{t+1}$ denotes the updated position of the *i*-th bird’s nest in the *t +* 1-th iteration, *α* is the factor of step size used to control the random walk step, the product ⨂ is a kind of calculation that denotes entry-wise multiplication, and Lévy(λ) is a random number, which is drawn from a Lévy distribution,

(2) $$Lévy\left(\lambda \right)\sim \mu =t^{-\lambda },\;1<\lambda \leq 3.$$

In the implementation, Lévy(λ) can be calculated as follows:

(3) $$Lévy\left(\lambda \right)\sim \frac{\varphi \mu }{{\left|v\right|}^{\frac{1}{\beta }}},$$

where μ and v follow a normal distribution, and β is a distribution factor between 0.3 and 1.99. Here, β=1.5; φ is expressed as follows:

(4) $$\varphi =\left\{\frac{г(1+\beta )sin\left(\frac{\pi \beta }{2}\right)}{г\left(\frac{1+\beta }{2}\right)\times \beta \times 2^{(\beta -1)/2}}\right\},$$

where г(·) is the gamma function.

α is expressed as

(5) $$\alpha =\alpha_{0}\left(x_{i}^{t}-x_{best}^{t}\right),$$

where $x_{best}^{t}$ denotes the optimal bird’s nest of the *t*-th iteration; $\alpha_{0}$ is the control parameter that controls the random step size and usually takes the value of 0.01.

Equation (1) can be reformulated as

(6) $$X_{i}^{t+1}=X_{i}^{t}+\alpha_{0}\frac{\varphi \mu }{{\left|v\right|}^{\frac{1}{\beta }}}\left(x_{i}^{t}-x_{best}^{t}\right).$$

### 2.3. Improved Cuckoo Search Algorithm

Although the CS algorithm is used in many fields with its outstanding advantages, it still has some drawbacks and shortcomings [45]. Firstly, the random initial population lacks uniformity of distribution. Secondly, the optimal solution is greatly affected by the fixed probability $P_{a}$ of the host bird finding the parasitic egg. Thirdly, the Lévy flight mechanism lacks adaptivity due to alternating large and small steps for the global search. Specifically, the lack of adaptive ability in local search will reduce the search efficiency and increase the search time, making it difficult to obtain a balance between precision and search ability.

Consequently, to address these issues, improvements were made in this paper in terms of both population diversity and parameter adaptation, respectively.

#### 2.3.1. Initial Population Chaoticization

Defined as highly unstable/unpredictable motion in finite phase space, chaos often occurs in deterministic nonlinear dynamical systems. According to the change in chaos behavior, chaos has randomness and strong ergodicity. Thus, we proposed a chaotic cuckoo initial population to improve the population diversity through chaotic mapping, while reducing the possibility of falling into local optimum at the initial stage. Tent chaotic mapping was used in this paper, which can be expressed as follows:

(7) $$x_{n+1}=\{\begin{array}{c} 2x_{n},\;0\leq x_{n}\leq \frac{1}{2}\\ 2\left(1-x_{n}\right),\;\frac{1}{2}\leq x_{n}\leq 1 \end{array}.$$

The specific implementation process is described below.

First, a random $x_{0}$ is generated, and then the *n* nest locations are obtained by iterating using Equation (7).

(8) $$x_{k,j}^{\left(i\right)}={\left[x_{k,1}^{\left(i\right)},x_{k,2}^{\left(i\right)},\cdots ,x_{k,n}^{\left(i\right)}\right]}^{T},\;k=C,\sigma ,$$

where *i* represents the iteration number, *j* represents the number of nests, and x∈(0, 1).

Then, the chaotic sequence values are mapped to the value range of SVR critical parameters.

(9) $$cx_{k,j}^{\left(i\right)}=Lb_{j}+x_{k,j}^{\left(i\right)}(Ub_{j}-Lb_{j}),$$

where *Lb* = 0.001; *Ub* = 500.

#### 2.3.2. Adaptive Control Parameters

The original CS algorithm adopts a fixed value for both $P_{a}$ and $a_{0}$. Typically, $P_{a}$ is 0.25, and $a_{0}$ is 0.01. However, in the face of practical different optimization problems, it is often necessary to adjust these parameters to suit specific needs on the basis of personal experience; hence, so it is a challenging task to choose the appropriate parameters $P_{a}$ and $a_{0}$ [44].

Due to the lack of control parameter adaptivity, the algorithm does not guarantee fast convergence. If $a_{0}$ is relatively large, it can improve the global search ability of the algorithm, but reduce the local search ability; if $a_{0}$ is too small, the local search is better, but the total number of iterations increases, and the convergence may become worse. Therefore, $a_{0}$ should gradually decrease with the iterative process of the algorithm. In addition, during the iterative search process, as the quality of cuckoo individuals gradually increases, the intensity of population evolution should be appropriately increased to avoid the algorithm falling into local optimal solutions; thus, $P_{a}$ should also gradually decrease with the iterative process. Therefore, to improve the performance of the CS algorithm, we propose an adaptive control parameter strategy, which is represented as follows:

(10) $$a_{0}=a_{min}+0.5\left(a_{max}-a_{min}\right)\left(cos\left(log\left(1+\frac{\left(e-1\right)t}{T}\right)\pi \right)+1\right),$$

(11) $$P_{a}=P_{amin}+\left(P_{amax}-P_{amin}\right){\left(\frac{200-t}{T}\right)}^{2},$$

where *t* and *T* are the number of the current iteration and the number of total iterations, respectively. The parameters in the adaptive control parameter strategy were set as follows: $a_{min}$ = 0.001; $a_{max}$ = 0.2; $P_{a,\;min}$ = 0.1; $P_{a,max}$ = 0.70; *T* = 200.

### 2.4. Architecture of the ICS-SVR Model

**Figure 2:**
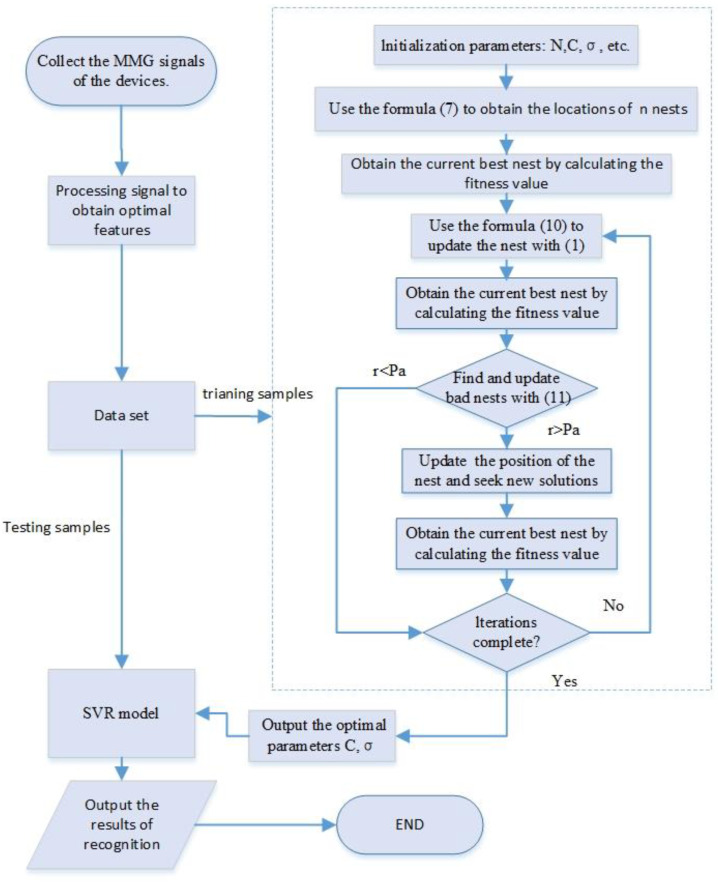
The procedure of ICS-SVR.

The designed ICS can quickly find the optimal solution without searching all the parameter points; accordingly, this method can greatly improve the efficiency of finding the optimal solution in a wide range of SVR parameters *C* and *σ*. The optimal parameters obtained can be used to improve the fitting performance of SVR. In this paper, a muscle force estimation model was constructed on the basis of ICS-SVR, and the procedure of ICS-SVR is illustrated in Figure 2.

### 2.5. Gray Correlation Analysis

Choosing the optimal features is crucial in pattern recognition because it determines the accuracy and generalization ability of classifiers and prediction models [46]. Therefore, it is a challenging task to extract effective features from the MMG signals that can reflect muscle activity. In practical engineering problems, since the data collected by the instrumentation are of great importance, it is not recommended to destroy the original structure of the data during modeling if not absolutely necessary.

Gray system theory was proposed by Deng Julong in 1982 on the basis of the mathematical theory of systems engineering, which is one of the important achievements in the field of uncertainty system research [47]. GRA is an important part of gray system theory, where a gray relationship is an uncertain relationship between two variables. The dominant factors are determined by calculating the correlation degree between multiple factors and the same reference sequence [48]. Therefore, in this paper, GRA was used to select the effective MMG features that are most associated with knee joint extension force. The specific implementation of GRA is described below.

Let F={F(k)|k=1,2,⋯,n} be the knee joint extension force sequence and $X_{i}=\{x_{i}\left(k\right)|k=1,2,\cdots ,n;\;i=1,2,\cdots ,m\}\;$be the MMG feature sequences.

Relational coefficients between the reference sequence (the knee joint extension force sequence) and comparison sequences (the MMG feature sequences) were calculated as follows:

(12) $$\xi_{i}\left(k\right)=\frac{\underset{i}{\min}\underset{k}{\min}\left|F\left(k\right)-x_{i}\left(k\right)\right|+\rho \underset{i}{\max}\underset{k}{\max}\left|F\left(k\right)-x_{i}\left(k\right)\right|}{\left|F\left(k\right)-x_{i}\left(k\right)\right|+\rho \underset{i}{\max}\underset{k}{\max}\left|F\left(k\right)-x_{i}\left(k\right)\right|}.$$

Let $\Delta_{i}\left(k\right)=\left|F\left(k\right)-x_{i}\left(k\right)\right|$; then, the difference sequence is

(13) $$\Delta_{i}\left(k\right)=\left(\Delta_{i}\left(1\right),\Delta_{i}\left(2\right),\cdots ,\Delta_{i}\left(n\right)\right).$$

After simplifying Equation (12), we can get

(14) $$\xi_{i}\left(k\right)=\frac{\underset{i}{\min}\underset{k}{\min}\Delta_{i}\left(k\right)+\rho \underset{i}{\max}\underset{k}{\max}\Delta_{i}\left(k\right)}{\Delta_{i}\left(k\right)+\rho \underset{i}{\max}\underset{k}{\max}\Delta_{i}\left(k\right)}.$$

A small resolution coefficient (*ρ*) indicates a great resolution. In general, the range of *ρ* is (0, 1). The resolution is best when *ρ* ≤ 0.5463; usually, *ρ* = 0.5.

The relational coefficient represents the value of the correlation degree between the knee joint extension force sequence and the MMG feature sequence.

Usually, the average of the correlation coefficient at each moment is quantitatively expressed as the relational degree between the knee joint extension force sequence and the MMG feature sequence. The relational degree $r_{i}$ is calculated as follows:

(15) $$r_{i}=\frac{1}{n}\overset{n}{\underset{k=1}{\sum }}\xi_{i}\left(k\right),\;k=1,2,\cdots ,n.$$

### 2.6. Performance of the Models

The performance evaluation indicators for the estimation models were the root-mean-square error (RMSE), mean absolute percentage error (MAPE), and correlation coefficient (*R*), which are defined as follows:

(16) $$\mathrm{RMSE}=\sqrt{\frac{1}{N}\overset{N}{\underset{i=1}{\sum }}{\left({\hat{y}}_{i}-y_{i}\right)}^{2}},$$

(17) $$\mathrm{MAPE}=\frac{1}{N}\overset{N}{\underset{i=1}{\sum }}\left|\frac{{\hat{y}}_{i}-y_{i}}{y_{i}}\right|,$$

(18) $$R=\frac{Cov({\hat{y}}_{i},y_{i})}{\sqrt{D\left({\hat{y}}_{i}\right)}\sqrt{D\left(y_{i}\right)}},$$

where ${\hat{y}}_{i}$ is the estimated value, $y_{i}$ is the actual value, D(·) is the calculated variance, Cov(·) is the covariance, and *N* is the number of samples in the test set. Generally speaking, the closer RMSE and MAPE are to 0 and *R* is to 1, the closer the estimated value of the model is to the actual value.

## 3. Signal Acquisition and Preprocessing

### 3.1. Experimental Procedure and Signal Processing

A total of five healthy male subjects aged 22–25 years without neuromuscular and musculoskeletal diseases were recruited and briefed on the experiment and the potential associated risks. Informed consent forms were signed by the subjects before experimentation. The subjects were asked to sit comfortably in a test chair with their right leg fixed and bent at a 90° angle. The MMG and the force signals were recorded using a DAQ device (VK702, VKinging, Inc., Shenzhen, China) at a sampling frequency of 1 kHz. MMG signals came from the acceleration sensor (ADXL335, Analog Devices, Inc., Norwood, MA, USA), while the force signals came from the force sensor (DYLF-30, 0–300 N, Bengbu Sensor, Inc., Bengbu, China). Three accelerometers were placed on the clothes and bound with elastic bandages to the muscle belly positions of the rectus femoris (RF), vastus lateralis (VL), and vastus medialis (VM) muscles, respectively. The data collection process was completed across two days. On the first day, subjects were familiarized with the experimental process and precautions, and maximal voluntary contraction force (MVC) was collected. On the second day, forces of 10–80% MVC and corresponding MMG signals were collected. Sufficient rest time (2–5 min) was provided between experiments to avoid muscle fatigue.

To attenuate the effects of movement and noise, the MMG signals were filtered by a fourth-order Butterworth filter with a passband of 5–100 Hz [49], and the force signals were filtered by a third-order Butterworth filter with a low passband of 2 Hz. Due to the fluctuation of force in the process of data collection, 6 s of stable muscle force data, whose fluctuation range was less than 5% of the relative value, were selected as the experimental data.

### 3.2. Feature Extraction

In this paper, sliding windows were used for feature extraction of MMG signals. The window length was 1000 data points, and the overlap length was 100 data points.

Feature extraction plays a key role in pattern recognition, which directly determines the accuracy of prediction and estimation. Therefore, in this paper, we extracted not only time-domain features, frequency-domain features, and time–frequency-domain features, but also nonlinear dynamics features. Specifically, root-mean-square (RMS), kurtosis, standard deviation (SD), slope sign change (SSC), mean absolute values (MAV), zero crossings (ZC), and waveform length (WL) were selected as time-domain features. Mean power frequency (MPF) and median frequency (MDF) were selected as the frequency-domain features. After a three-layer wavelet packet decomposition, wavelet packet energy (WPE) and the energy of every frequency band (WP1 WP2, WP3, WP4, WP5, WP6, WP7, WP8) were calculated as time–frequency-domain features. Lempel–Ziv complexity (LZC), sampling entropy (SampEn), approximate entropy (ApEn), fuzzy entropy (FuzzyEn), distribution entropy (DistEn), box-counting fractal dimension (FD), and largest Lyapunov exponent (LyapExp) were calculated as nonlinear dynamics features. The details of the specific nonlinear dynamics feature extraction method can be found in [50,51,52,53,54]. As a result, we extracted 25 dimensional features from each segment, with a total of 75 dimensional features for the three channels.

Additionally, for eight different strength forces, a sample dataset was obtained for each subject, resulting in a total of 400 features. To further train and test the model, the 400 samples were randomly shuffled; 90% of them were selected as the training set and 10% were selected as the test set.

### 3.3. Data Normalization

To eliminate the impact of the value range or uneven magnitude of different features, the MMG features and muscle force were normalized using the Z-score method, which can be expressed as follows:

(19) $$z=\frac{x-\bar{x}}{\sigma },$$

where $\bar{x}=\frac{1}{n}\overset{n}{\underset{i=1}{\sum }}x$, and $\sigma =\sqrt{\frac{1}{n-1}\overset{n}{\underset{i=1}{\sum }}\left(x-\bar{x}\right)}$. The overfitting of the ICS-SVR model can be avoided to some extent by normalizing the data.

## 4. Experiments and Results

In this paper, our experiments consisted of three parts. In the first part, test benchmark functions were used to test the performance of different swarm intelligence algorithms. If ICS was shown to have better search performance than other algorithms, it could be used to optimize SVR critical parameters. In the second part, the relational degree between MMG features and knee joint extension force was calculated to obtain different feature combinations, which were input into the ICS-SVR model to obtain the estimated knee extension force. Then, the optimal feature combination sequence was obtained by comparing and analyzing the estimation accuracy under different feature combinations. In the third part, the ICS-SVR model was compared with other classical models in the estimation of the knee joint extension force. If the estimation results of the model were found to be closer to real data than other models, the model could be considered the best.

### 4.1. Performance Analysis of ICS Algorithm

To verify the effectiveness of the designed ICS algorithm, a comparative simulation experiment was designed to test the optimization performance.

Performance analysis was performed on the ICS algorithm and other algorithms, such as particle swarm optimization (PSO), gray wolf optimizer (GWO), and the original CS algorithm, using the four global optimal test benchmark functions without constraints. Parameter values in the four optimization algorithms are shown in Table 1. The four test benchmark functions are expressed below.

(1) Rosenbrock function:

(20) $$f_{1}=\overset{n-1}{\underset{i=1}{\sum }}\left[100{\left(x_{i+1}-x_{i}^{2}\right)}^{2}+{\left(x_{i}-1\right)}^{2}\right],-2.048\leq x_{i}\leq 2.048.$$

(2) Griewank function:

(21) $$f_{2}=\overset{n}{\underset{i=1}{\sum }}\frac{x_{i}^{2}}{4000}-\overset{n}{\underset{i=1}{\prod }}\cos\left(\frac{x_{i}}{\sqrt{i}}\right)+1,-600\leq x_{i}\leq 600.$$

(3) Cross-in-tray function:

(22) $$f_{3}=-0.0001{\left(\left|\sin\left(x_{i}\right)\sin\left(x_{i+1}\right)\exp\left(\left|100-\frac{\sqrt{x_{i}^{2}+x_{i+1}^{2}}}{\pi }\right|\right)\right|+1\right)}^{0.1},-10\leq x_{i}\leq 10.$$

(4) Schaffer function:

(23) $$f_{4}=0.5+\frac{{\left(\sin\sqrt{x_{i}^{2}+x_{i+1}^{2}}\right)}^{2}-0.5}{{\left[1+0.01\left(x_{i}^{2}+x_{i+1}^{2}\right)\right]}^{2}},-10\leq x_{i}\leq 10.$$

**Figure 3:**
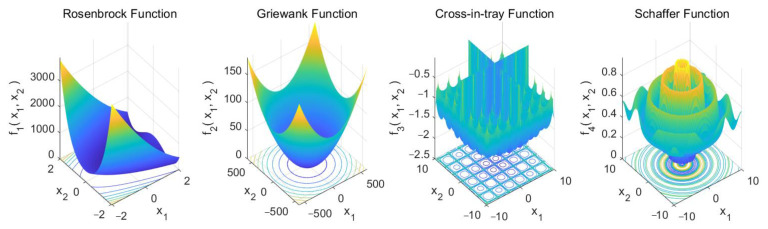
The search space of the four test benchmark functions.

The Rosenbrock function, also known as the valley or banana function, has a global minimum $f_{1}\left(X^{*}\right)=0$ at $X^{*}=\left(1,\ldots ,1\right)$. The Griewank function has many widespread and regularly distributed local minima and a global minimum $f_{2}\left(X^{*}\right)=0$ at $X^{*}=\left(0,\ldots ,0\right)$. The cross-in-tray function has a global minimum $f_{3}\left(X^{*}\right)=-2.06261$ at four points of $X^{*}=\left(\pm 1.3491,\;\pm 1.3491\right)$. The Schaffer function has a strongly oscillating behavior, with many local minima and a global minimum $f_{4}\left(X^{*}\right)=0$ at $X^{*}=\left(0,\ldots ,0\right)$ [45,55,56]. The search space of the four test benchmark functions is shown in Figure 3.

**Figure 4:**
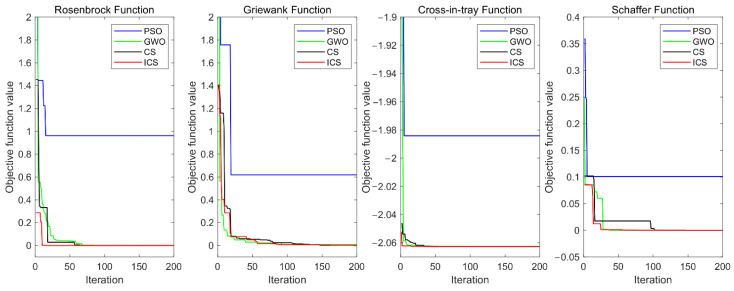
Comparison of convergence curves of the four algorithms for the four test benchmark functions.

The purpose of the test is to find the global minimum of all four functions. The PSO algorithm, the GWO algorithm, the original CS algorithm, and the ICS algorithm were terminated when the number of iterations reached 200. The convergence curves are shown in Figure 4. As can be seen from Figure 4, the ICS algorithm could converge to or was much closer to the optimal solution in a shorter time and was more accurate than the other algorithms in the four test benchmark functions. That is to say, the proposed new algorithm could effectively improve the convergence speed and convergence accuracy in search attempts.

To eliminate the difference in each experiment, the four algorithms were executed 50 times each. The numerical results of the selected indices, including the optimal solution, worst solution, average solution, and SD, are presented in Table 2.

Comparing the results presented in Table 2, it can be obviously seen that they almost always succeeded in finding the global minimum; however, the ICS algorithm was superior to PSO, GWO, and CS in all indices. In general, it can be concluded that the ICS algorithm contributed to superior performance, while CS and GWO were better than PSO, and PSO performed the worst. Therefore, the designed ICS algorithm was used in this paper to optimize the critical parameters of SVR.

### 4.2. Feature Combination Sequence Selection with GRA

**Figure 5:**
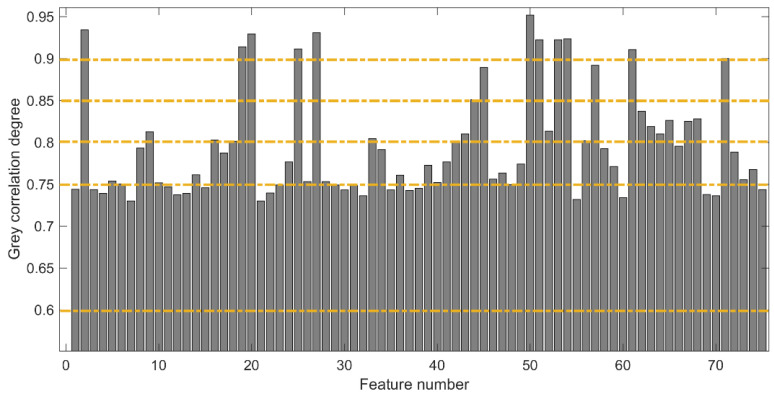
GRA analysis of MMG features of subject S1.

A total of 25 features were extracted from the MMG signal segment of each channel, and a total of 75 features were obtained from the three channels. Among these features, there were many unrelated or low-correlation features, and inputting all of these features into the model would lead to overfitting and a heavy computational burden. Therefore, in this paper, GRA was used for MMG feature selection to extract features exhibiting a high correlation with knee joint extension force. Firstly, based on GRA, the relational degree between knee joint extension force and various MMG features was calculated, and the results are shown in Figure 5. Usually, the classification of the relational degree is as follows: 0.8–1 indicates a strong correlation, 0.6–0.8 indicates a general correlation, and 0.6–0 indicates a weak correlation. To obtain the optimal features to describe the knee extension force, we selected the features with a correlation of 0.6–1 as feature combination A, features with a correlation of 0.75–1 as feature combination B, features with a correlation of 0.8–1 as feature combination C, features with a correlation of 0.85–1 as feature combination D, and features with a correlation of 0.9–1 as feature combination E, and we input them into the ICS-SVR model for knee joint extension force estimation; the results are presented in Table 3.

Generally, a smaller value of RMSE and MAPE and a larger value of R indicate a better effect of the feature combination of knee joint extension force estimation. From Table 3, it can be seen that feature combination D, i.e., the feature combination with a relational degree greater than 0.85, had the best results for knee joint extension force estimation with the smallest RMSE, the smallest MAPE, and the largest *R*. Therefore, the best estimation effect was obtained by selecting feature combination D, i.e., the selected MMG feature sequence was 2, 19, 20, 25, 27, 44, 45, 50, 51, 53, 54, 57, 61, and 71. This also indicates that different feature combinations had a certain influence on the estimation of knee joint extension force, and some features did not correlate well with the estimated results.

**Figure 6:**
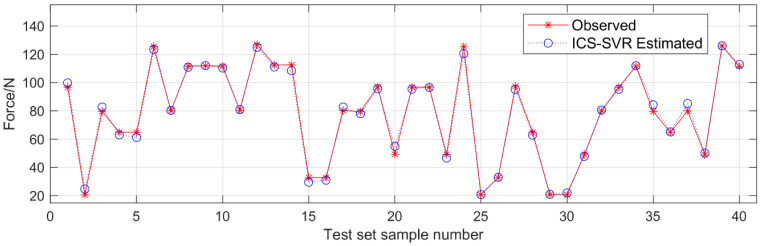
Results of knee joint extension force estimation for subject S1 with feature combination D.

As a result, in the process of muscle force estimation, the feature sequence of feature combination D was selected in this paper to avoid the overfitting of the model caused by too many input features. Figure 6 shows the results of muscle force estimation for subject S1 with feature combination D. As can be seen in Figure 6, the estimated values were mostly consistent with the actual observed values, indicating that the proposed method is effective.

To further verify the validity of the proposed method, muscle force estimation was performed in five subjects. The results of the knee joint extension force estimation for the five subjects are shown in Table 4. The results indicate that muscle force estimation using feature combination D in the five subjects experiment outperformed the effect of the other feature combinations.

### 4.3. Comparative Performance of the Proposed Model with Classical Machine Learning Models

To further demonstrate the validity of the ICS-SVR model, a comparison was made with BPNN, ELM, and CS-SVR. The network structures of BPNN and ELM were 14–10–1 and 14–8–1, respectively. The estimated results of subject S1 upon executing the different models 10 times are shown in Table 5. As shown in Table 5, the proposed model outperformed BPNN, ELM, and CS-SVR in terms of estimation effect. Additionally, compared with the fluctuation of their estimated results, the proposed model had the least fluctuation and the most stable estimation results.

**Figure 7:**
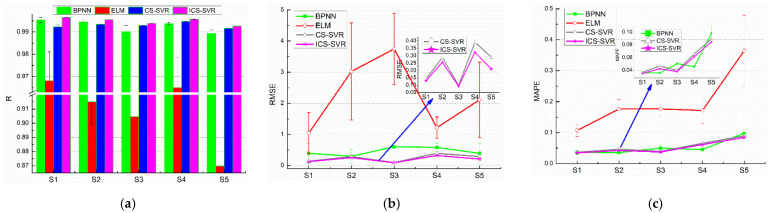
Statistical analysis of knee joint extension force estimation with different regression models. (**a**) The *R*-value of the force estimation against the actual observed values. (**b**) The RMSE of the force estimation against the actual observed values. (**c**) The MAPE of the force estimation against the actual observed values.

Furthermore, different models were used to estimate muscle force for the five subjects. The results of knee joint extension force estimation are shown in Figure 7 and Table 6. As can be seen in Figure 7, the force estimation effects of each subject with ICS-SVR were superior to those with BPNN, ELM, and CS-SVR. In addition, using the proposed model for muscle force estimation, all five subjects obtained outstanding average results with the highest *R* values and the lowest RMSE and MAPE, as shown in Table 6. In particular, compared with BPNN, ELM, and CS-SVR models, the estimated results of the proposed model had less fluctuation. Meanwhile, in terms of numerical results, the accuracy of the muscle force estimation of the proposed model in this paper was optimal compared with the results of the literature [25,26,27,28,29]. Furthermore, it is shown that our proposed model had unparalleled applicability and prediction accuracy in knee joint extension force estimation.

## 5. Discussion

The human body often needs to exert force during various movements, which play an important role in daily activities. To achieve flexible human–computer interaction during effective training with rehabilitation devices, this study aimed to decode human movement intentions through muscle interfaces to achieve the estimation of interaction forces during isometric contractions. Muscle force estimation can be used to improve the naturalness and flexibility of human–machine coordinated movement. Many previous studies used sEMG signals for human movement classification and kinetic parameter estimation. However, sEMG detection has some disadvantages compared with MMG signals, which brings many inconveniences to practical applications. Therefore, decoding human intentions using MMG signals can provide a more natural and portable human–computer interaction, which has attracted the attention of a wide range of researchers. Furthermore, MMG signals can objectively reflect muscle activity and are expected to be widely used in rehabilitation equipment, powered prosthesis, etc.

The muscle contraction part of the kinematic chain through the limb effector can generate forces that interact with the environment [57]. Due to the anthropotomy and motion control principle, the excitation–contraction of muscles will be dynamic and coupled. Therefore, it is challenging to estimate muscle force accurately and efficiently. In this paper, the relationship between knee joint extension force and MMG was established through GRA and ICS-SVR. Our proposed combined scheme of GRA and ICS-SVR could better deal with the difficulty of MMG feature selection and achieve accurate knee joint extension force estimation.

For the estimated models, if excessive MMG features are input, the training speed and efficiency of the estimation models would be reduced, and the convergence may not be obtained. Therefore, it is worth noting that a suitable feature sequence for the force estimation is an issue that must be considered. The main principle in selecting MMG features is the effectiveness of the force estimation. The relational degree between the knee joint extension force sequence and the MMG feature sequence calculated by GRA facilitates the easy selection of related features that are highly correlated with the reference sequence, thus effectively avoiding the difficulty of feature selection and the long time needed to test the selected features. Therefore, GRA can be performed on the MMG features to fulfill the above effectiveness, avoiding the destruction of the data and maintaining the physical meaning of the original data. From the experimental results, it can be concluded that the optimal feature combination obtained using GRA had a better estimation effect than all feature combinations, whereby RMSE was reduced by 25.40%, MAPE was reduced by 13.83%, and MAPE was improved by 0.03%.

According to the findings of a previous study, SVR is an effective method for muscle force estimation, but its effect is extremely susceptible to critical parameters. Therefore, we proposed an ICS method to optimize the critical parameters of SVR. Compared with PSO, GWO, and CS, the designed ICS algorithm was optimal in terms of search performance. The adaptability of the CS algorithm was enhanced by using a chaotic optimization initial population and adaptive control parameters, such that the critical parameters searched became more suitable for MMG characteristics. Subsequently, to demonstrate the validity of the model, we compared the proposed ICS-SVR with classical machine learning algorithms, such as BPNN, ELM, and CS-SVR, as a function of specific knee joint extension force estimation cases. The results showed that the proposed ICS-SVR model performed optimally in knee joint extension force estimation. In addition, the overall average results were about the same compared to the pre-IGWO-SVR model results, but obtained a nice performance improvement in RMSE, from 0.2492 to 0.2205. The features in the previous study were selected empirically, while the present study was based on GRA for automatic selection, which is more adaptable to complex time-series MMG signals; this result can perhaps bring immeasurable effect in subsequent muscle force estimation under complex conditions. Further research is needed to apply the proposed scheme to the muscle force estimation under complex conditions based on MMG signals and to the estimation of movement parameters and muscle force based on other time-series signals, such as sEMG and EEG.

MMG can be used as a useful tool to examine various conditions of muscle activity. Consequently, it can be applied in many fields such as muscle function assessment, power prosthesis, and medical rehabilitation [5,10]. Usually, muscle atrophy or muscle damage may occur as a result of injury or disease, and muscle weakness may occur. The main purpose of rehabilitation training is to restore normal function to the atrophied muscles. To facilitate an effective rehabilitation process, the relevant equipment should provide the appropriate training intensity according to the patient’s muscle activity to provide the required assistance. Nevertheless, one of the most important training processes is muscle isometric contraction. To make this process more effective, MMG can be used to monitor the degree of muscle activation and muscle force interaction with the external environment, which can help to provide an understanding of muscle function and recovery effects, and further provide a reasonable training program.

In this paper, a high estimation accuracy was achieved in the knee extension force estimation. In addition to the fact that this result only applies to the muscle isometric contraction case, this study had some limitations, such as limited sample size and single physical health status.

## 6. Conclusions

In this paper, a combined scheme of GRA and ICS-SVR was developed to accurately estimate muscle force from MMG signals. The method screened out the features reflecting muscle activity by GRA and reduced the input features of the model, thereby effectively improving the efficiency of the muscle force estimation model. Meanwhile, the CS algorithm was improved by a chaotic optimization initial population and adaptive control parameters, resulting in a further improvement of the global search performance. Finally, ICS was applied to the critical parameter search of SVR to obtain excellent muscle force estimation effects. In particular, the mean values of RMSE and MAPE obtained by ICS-SVR were significantly lower than those obtained by BPNN, ELM, and CS-SVR, and the mean value of *R* was closer to 1. In conclusion, the proposed combined scheme of ICS-SVR and GRA is a feasible, effective, and promising method for the estimation of knee joint extension force, which is also expected to further improve the working performance of related devices. Future research will be focused on experiments on dynamic force generation during isometric muscle contraction and force generation during joint movement to further refine key interaction techniques required for specific rehabilitation and powered prostheses.

## Figures and Tables

**Table 1:** Parameter values in the four optimization algorithms with a population size of 20.

Algorithm	Parameter
PSO	c1 = c2 = 1, k = 0.5, wV = 0.9, wP = 0.9
GWO	r1, r2 ∈ (0, 1), a ∈ (0, 2)
CS	$P_{a}=0.25$ , β=1.5 $,\;a_{0}=0.01$
ICS	β=1.5

PSO = particle swarm optimization algorithm; GWO = gray wolf optimization algorithm; CS = cuckoo search algorithm; ICS = improved cuckoo search algorithm.

**Table 2:** Results of optimization algorithms for solving four benchmark functions with D = 2.

Test Function	Algorithm	Optimal Solution	Worst Solution	Average Solution	SD
Rosenbrock function	PSO	0.0142	3.9689	1.0774	1.0380
	GWO	4.377 × 10^−7^	2.503 × 10^−4^	2.383 × 10^−5^	4.341 × 10^−5^
	CS	1.389 × 10^−13^	3.605 × 10^−4^	7.822 × 10^−6^	5.093 × 10^−5^
	**ICS**	**1.816 × 10^−13^**	**9.313 × 10^−7^**	**4.922 × 10^−8^**	**1.541 × 10^−7^**
Griewank function	PSO	0.3838	13.0372	1.7129	1.8178
	GWO	0	0.0271	0.0054	0.0056
	CS	9.678 × 10^−7^	0.0089	0.0044	0.0031
	**ICS**	**2.618 × 10^−6^**	**0.0081**	**0.0025**	**0.0029**
Cross-in-tray function	PSO	−2.0622	−1.8755	−2.0260	0.0438
	GWO	−2.0626	−2.0626	−2.0626	1.257 × 10^−7^
	CS	−2.0626	−2.0626	−2.0626	6.519 × 10^−11^
	**ICS**	**−2.0626**	**−2.0626**	**−2.0626**	**3.352 × 10^−11^**
Schaffer function	PSO	0.0113	0.2443	0.0940	0.0391
	GWO	0	0.0851	0.0204	0.0367
	CS	4.749 × 10^−7^	0.0851	0.0019	0.0120
	**ICS**	**2.113 × 10^−9^**	**0.0085**	**6.824 × 10^−4^**	**0.0016**

PSO = particle swarm optimization algorithm; GWO = gray wolf optimization algorithm; CS = cuckoo search algorithm; ICS = improved cuckoo search algorithm.

**Table 3:** Estimated results of different feature combinations of subject S1.

Combination	RMSE	MAPE	*R*
Feature combination A	0.1768	0.0405	0.9963
Feature combination B	0.7493	0.0377	0.9954
Feature combination C	0.1611	0.0416	0.9946
Feature combination D	**0.1319**	**0.0349**	**0.9966**
Feature combination E	0.4282	0.0327	0.9957

RMSE = root-mean-square error; MAPE = mean absolute percentage error; *R* = correlation coefficient.

**Table 4:** Results of the knee joint extension force estimation for the five subjects with feature sequences of different feature combinations.

Combination	RMSE ± SD	MAPE ± SD	*R* ± SD
Feature combination A	0.7511 ± 0.7645	0.0550 ± 0.0120	0.9937 ± 0.0042
Feature combination B	0.6012 ± 0.3840	0.0604 ± 0.0190	0.9912 ± 0.0046
Feature combination C	0.6706 ± 0.5202	0.0649 ± 0.0241	0.9920 ± 0.0026
Feature combination D	**0.2761** **± 0.2396**	**0.0522** **± 0.0208**	**0.9949** **± 0.0016**
Feature combination E	0.8214 ± 0.6718	0.0644 ± 0.0464	0.9916 ± 0.0059

Values are the mean ± SD.

**Table 5:** Comparison of the estimation results for S1 using different models.

Model	RMSE	MAPE	*R*
BPNN	0.3952 ± 0.3246	0.0361 ± 0.0064	0.9954 ± 0.0012
ELM	1.0464 ± 0.6673	0.1071 ± 0.0190	0.9681 ± 0.0130
CS-SVR	0.1424 ± 0.0274	0.0358 ± 0.0026	0.9923 ± 0.0008
ICS-SVR	**0.1295** **±** **0.0021**	**0.0349** **±** **8.06 × 10^−6^**	**0.9966** **±** **1.38 × 10^−6^**

BPNN = backpropagation neural network; ELM = extreme learning machine; CS-SVR = support vector regression optimized by cuckoo search algorithm; ICS-SVR = support vector regression optimized by the improved cuckoo search algorithm.

**Table 6:** Comparison of the estimation results for the five subjects using different models.

Model	RMSE	MAPE	*R*
BPNN	0.4706 ± 0.1299	0.0575 ± 0.0241	0.9919 ± 0.0022
ELM	2.5252 ± 0.9507	0.2223 ± 0.0822	0.9137 ± 0.0341
CS-SVR	0.2673 ± 0.1061	0.0603 ± 0.0193	0.9932 ± 0.0011
ICS-SVR	**0.2205** **±** **0.0840**	**0.0565** **±** **0.0185**	**0.9945** **±** **0.0013**

Values are the mean ± SD.

## Data Availability

The data used to support the findings of this study are included within the article.
